# A Proposed Target Product Profile and Developmental Cascade for New Cryptosporidiosis Treatments

**DOI:** 10.1371/journal.pntd.0003987

**Published:** 2015-10-08

**Authors:** Christopher D. Huston, Thomas Spangenberg, Jeremy Burrows, Paul Willis, Timothy N. C. Wells, Wesley van Voorhis

**Affiliations:** 1 Department of Medicine, University of Vermont College of Medicine, Burlington, Vermont, United States of America; 2 Medicines for Malaria Venture, Geneva, Switzerland; 3 Department of Medicine, University of Washington School of Medicine, Seattle, Washington, United States of America; University of California San Diego School of Medicine, UNITED STATES

The Global Enteric Multicenter Study (GEMS), a study of infectious diarrhea involving over 20,000 children at seven sites in sub-Saharan Africa and South Asia, recently reported that the little-studied protozoan parasite *Cryptosporidium* ranks second to rotavirus as a cause of life-threatening diarrhea in infants and was also associated with growth stunting and excess mortality [1]. *Cryptosporidium* species, predominantly *Cryptosporidium hominis* and *Cryptosporidium parvum*, were previously well known for causing chronic diarrhea in AIDS patients, as well as for their chlorine resistance and their association with waterborne outbreaks in the developed world [2–4]. Numerous smaller studies had also demonstrated the importance of cryptosporidiosis in young children and its association with malnutrition (reviewed in [5]) [5–19], but *Cryptosporidium* had not previously garnered significant attention from the pharmaceutical industry or major funding organizations such as the Bill and Melinda Gates Foundation. The GEMS put cryptosporidiosis into context amongst more studied diarrheal pathogens and brought it to the attention of these organizations. No vaccine for cryptosporidiosis exists, and the available treatments for those most at risk are inadequate. The only licensed drug, nitazoxanide, is unreliable in severely malnourished children (~56% improvement in diarrhea at 7 days versus 26% in controls [20]), and shows no benefit relative to placebo in HIV-infected patients [20–22]. More reliable, efficacious, and faster-acting treatments are needed for these populations.

Interest in *Cryptosporidium* drug development has correspondingly increased, and despite a limited experimental system, several new molecules with in vitro and, in some cases, validated in vivo activity against *Cryptosporidium* have been reported [23–35]. However, data regarding these compounds are difficult to compare due to use of differing in vitro and in vivo models and lack of assay standardization (see [36] for a thorough review of this and other barriers). A major current challenge then is to develop a common framework to help *Cryptosporidium* researchers and funding agencies to prioritize screening hits and leads for further development and to define essential developmental milestones. In this article, we propose a target product profile (TPP) (Table 1) and testing cascade (Fig 1) that can be used to guide this process. This TPP will undoubtedly require revision as progress is made, but can provide clear initial goals and a framework for go/no-go decision making in order to facilitate the appropriate distribution of effort and limited resources.

**Fig 1:**
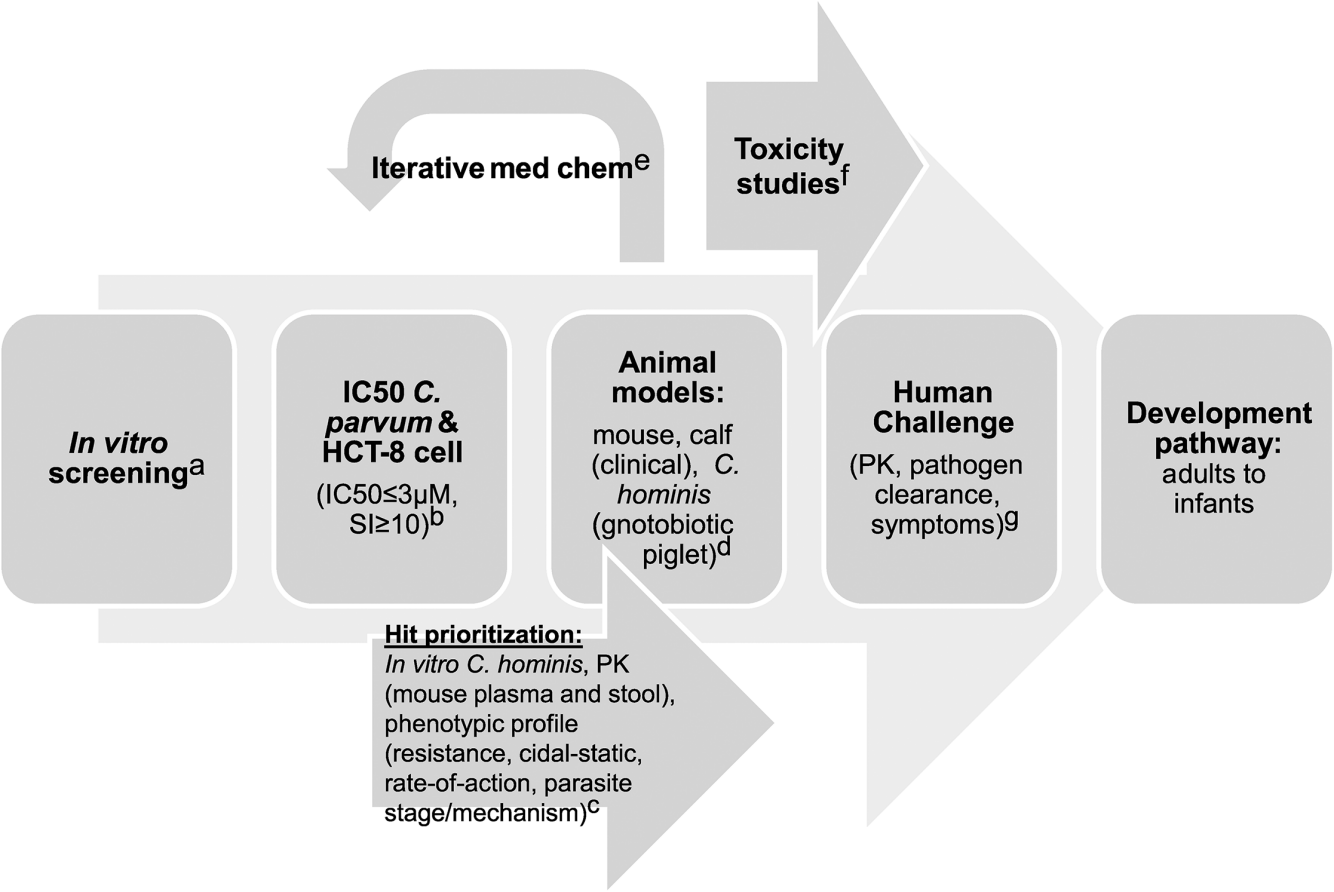
Proposed development scheme and anticipated costs for *Cryptosporidium* drug development. Abbreviations: pharmacokinetic (PK), 50% inhibitory concentration (IC50), selectivity index (SI, defined as IC50_host cell line_/IC50_*Cryptosporidium*_), and compound (cmpd). a. Screening: US$2–US$5 per compound; b. IC50s: US$100 per compound; c. Hit prioritization and mouse PK: US$5,000 per compound; d. Mouse: US$10K–US$20K/compound, Calves: US$50K/compound, Gnotobiotic piglet: US$200K/compound; e. Medicinal chemistry: US$500K/year x 3 years; f. Good laboratory practice (GLP) Toxicology Studies: US$1.65M; g. Human challenge: US$400K/compound.

**Table 1:** Proposed target product profile for treatments for diarrhea due to cryptosporidiosis.

Variable	Minimum essential	Ideal
**Indication**	Treatment of HIV-negative children aged 6–24 months and adults with diarrhea due to *Cryptosporidium hominis* or *Cryptosporidium parvum* infection	Treatment of children ≥ 1 month old and adults, including HIV-positive patients, with diarrhea due to cryptosporidiosis. Curative for additional diarrheal pathogens, and safe for use in syndromic treatment of diarrhea.
**Product**	Single agent or combination drug regimen	Single agent therapy
	Note that the risk of resistance is unknown and may require combination therapy.	
**Target populations**	Children ages 6–24 months with diarrhea due to cryptosporidiosis	Children ages 1–24 months with diarrhea due to cryptosporidiosis
	Immunocompetent adults with diarrhea due to cryptosporidiosis	Immunocompromised patients with diarrhea due to cryptosporidiosis
		Note that immunocompetent and immunocompromised patient populations may require distinct therapies.
**Target countries**	Countries that have been shown to have significant endemic cryptosporidiosis or that contribute heavily to the diarrhea burden in children	Countries accounting for 90% of morbidity and mortality due to diarrhea.
**Clinical efficacy**	Superiority to nitazoxanide in malnourished children	Cessation of diarrhea within 2 days in well nourished, HIV-negative children
	Equivalent to nitazoxanide in immunocompetent adults	≥90% efficacy in all patient populations
		Elimination of the effects of *Cryptosporidium* infection on malnutrition
**Microbiologic efficacy**	Superiority to nitazoxanide in malnourished children	Elimination of fecal parasite shedding within 2 days of starting therapy for all patient populations
	Equivalent to nitazoxanide in immunocompetent adults	
	Active against both *C*. *hominis* and *C*. *parvum*	
**Safety/drug-drug interactions**	Safe in patients ≥6 months old	Safe for syndromic treatment of diarrhea in patients ≥1 month old
	SAE rate ≤5% by Common Terminology Criteria for AEs; AEs ≥ Grade 2 no more than 30%	No drug-related SAEs by Common Terminology Criteria; minimal drug-related AEs
	No unmanageable drug–drug interactions	No CYP3A4 inhibition; no interactions with antiretroviral drugs
**Formulations and dosage**	Oral; maximum 3x/day for 14 days; liquid formulation or compatible with hydrodispersible tablet or granules appropriate for children available	Oral liquid or hydrodispersible tablet or granules given as a single dose
		Minimal or no food effect
**Stability**	≥2 years in Zone IVb (30°C 75% humidity)	≥3 years in Zone IV
**Total cost per patient**	$US2.00	≤$US0.50 (approximate total cost of nitazoxanide 100 mg/5 ml liquid formulation in India)

AE, adverse event; SAE, severe adverse event

## Essential Characteristics for Cryptosporidiosis Treatments

Nitazoxanide is the only drug with any validated clinical efficacy for treatment of human cryptosporidiosis, and it is FDA-approved for treatment of cryptosporidiosis in immunocompetent adults and children at least 1 year of age. In immunocompetent adults and less severely ill children, clinical response rates to nitazoxanide as high as 96% have been reported [37], and newer studies have suggested that it is safe in children less than 1 year of age [38]. Thus, although clearly inadequate for severely malnourished children and immunocompromised individuals [20], nitazoxanide can serve as a standard of care against which potential new treatments must be measured. Quantitative end points are essential to aid in comparison of different drug candidates. It is also useful to divide the goals for *Cryptosporidium* drug development into those required of a minimally acceptable treatment regimen and those that describe an ideal regimen. Table 1 summarizes the proposed minimum and ideal characteristics for new anti-*Cryptosporidium* therapeutic regimens.

Improved treatments for cryptosporidiosis are needed for two main target populations. Cryptosporidiosis has been repeatedly shown to be an important cause of diarrhea and to be associated with malnutrition in young children [1,5–19]. The GEMS, a case-control study conducted at 7 sites in Africa and South Asia, was the most comprehensive study to date [1]. In the GEMS, *Cryptosporidium* species were major contributors to life-threatening diarrhea in infants (0–11 months) and toddlers (12–23 months), ranking second and third overall in these age groups amongst 17 diarrheal pathogens studied [1]. Cryptosporidiosis accounted for over 10% of cases in infants and was the only pathogen significantly associated with increased mortality in toddlers. On the other hand, for children ages 24–59 months, *Cryptosporidium* was much less important. Collectively, the GEMS and earlier studies indicate that the greatest need for improved cryptosporidiosis treatments is for children aged 1–23 months with moderate-to-severe diarrhea or persistent diarrhea (diarrhea lasting longer than 14 days). The other major need is for treatments for HIV-positive children and adults with advanced AIDS and chronic diarrhea. *Cryptosporidium* may cause as much as 75% of chronic diarrhea in this patient cohort [18].

Despite these needs, the initial safety and efficacy studies for new anti-*Cryptosporidium* drugs will most likely be conducted first in immunocompetent adults, due to the complexity and ethics of conducting clinical trials in children. Development and regulatory approval is more complex for children <6 months old, as a result of differences in drug metabolism, a need to rely on sparse pharmacokinetic (PK) sampling, and difficulty of monitoring for side effects in very young children. Use of a human challenge model of infection and a head-to-head comparison with nitazoxanide is likely the most straightforward and cost-effective developmental path, since early-stage development in an endemic setting would be complicated by confounding effects from the frequent presence of multiple pathogens [39]. For this, a high-dose human challenge model with a high attack rate would be required. This approach would implicitly require efficacy at least equivalent to that of nitazoxanide in immunocompetent adults prior to moving on to more expensive field studies in young children and immunocompromised individuals, for whom the primary goal would be clinical superiority to nitazoxanide with reduced mortality as a secondary endpoint. Therefore, a minimum product requirement is efficacy equivalent to nitazoxanide for immunocompetent adults and superiority to nitazoxanide for treatment of malnourished children aged between 6–24 months. An ideal product would obviously be safe and effective for cryptosporidiosis diarrhea in all patient populations, including children as young as 1 month of age and immunocompromised individuals. Finally, although no causal relationship has been proven between *Cryptosporidium* infection and malnutrition, the strong association between the two suggests that an effective treatment for cryptosporidiosis might impact nutrition, along with physical and cognitive development. Studies of a drug’s effect on malnutrition would by necessity be of long duration, which argues against targeting malnutrition as a minimal goal, but the effects of treatments on malnutrition would be an important secondary end point.

*Cryptosporidium* treatments can theoretically provide case-by-case relief from the symptoms of cryptosporidiosis and reduce the spread of disease by accelerating elimination of parasite shedding. Thus, both endpoints should be considered. A minimal clinical goal would be equivalence to nitazoxanide with regards to resolution of diarrhea in well-nourished, HIV-negative children and adults, and superiority to nitazoxanide in malnourished children. Clinical efficacy should be evaluated to capture both the rate of improvement and the response rate, such as response (i.e., reduced diarrhea) and resolution rates following 24 hours and 7 days of treatment. An ideal product would result in rapid resolution of diarrhea (e.g., 2 days may be an achievable but lofty goal), and show improved clinical efficacy compared with nitazoxanide in all patient populations, including HIV-positive patients. Again, a minimally acceptable level of microbiologic efficacy would be superiority to nitazoxanide in severely malnourished children, which eliminates parasite shedding within 7 days of beginning treatment in ~52% of HIV-negative children as measured by modified acid fast staining of the feces [20]. Evidence of microbiologic efficacy in HIV-positive individuals would obviously be desirable. The relationship of parasite shedding to patient symptoms is not clear, but since more rapid elimination of parasite shedding is likely to reduce the transmission of disease, an ideal product would eliminate parasite shedding as rapidly as possible and, minimally, more quickly than nitazoxanide (i.e., within 7 days of initiating therapy) in all patient populations. These studies should utilize quantitative assays of fecal shedding, and based on superior sensitivity, we favor the use of a quantitative fecal PCR method, of which many have been developed [40–46].

Most human cryptosporidiosis is caused by either *C*. *hominis* or *C*. *parvum*, and it is essential that new drugs be effective against both species, especially since readily available diagnostic tests do not differentiate between species. Despite the paucity of differences in the published *Cryptosporidium* genome databases [47,48], all hits should be tested early during development in an intestinal epithelial cell infection model for selective activity against both parasites. This step has been omitted in many of the analyses of new hits and leads against *Cryptosporidium* because of the challenges involved in obtaining infectious *C*. *hominis* oocysts for experimental work (see Table 2). Little is known about variability in drug susceptibility amongst different *Cryptosporidium* clinical isolates, and efforts to test drug leads in vitro against a range of clinical isolates should ideally also be incorporated at an early stage of development. Prioritization of screening hits is further complicated because of variations in the assay methods and parasite isolates used by different laboratories. To aid in making go/no-go decisions for new chemical entities and decision making about the optimal distribution of resources, it is essential to compare new chemical series using standardized methods. This has been facilitated for malaria drug development by providing these basic assays as a service and establishing cutoffs for acceptable in vitro potency and selectivity. In the case of *Cryptosporidium*, reasonable initial cellular potency and selectivity cutoffs would be a 50% inhibitory concentration (IC50) ≤ 3 μM and a 10-fold window of selectivity against the HCT-8 cell line that is most widely used. Of note, this cutoff is lower than the measured potency of nitazoxanide (IC50 ~3.7 μM) [34]; however, use of a higher cutoff risks having resources focused on nonselective compounds with marginal activity, and large-scale phenotypic screening efforts have been initiated that are anticipated to yield an adequate number of screening hits (Case McNamara, California Institute for Biomedical Research [CALIBR], personal communication [see Acknowledgments section]). Depending on screening results, more or less stringent cutoffs could be adopted.

**Table 2:** Key needs to accelerate *Cryptosporidium* drug development.

• Validated developmental cascade (potential scheme in Fig 1)
• Selection of optimal animal models (rodent model [which one?], calf model, gnotobiotic piglet [*C*. *hominis*])
• Assays for hit prioritization
• Improved access to *C*. *hominis* oocysts
• Low-cost, robust animal model for *C*. *hominis*
• Knowledge of optimal pharmacokinetic/pharmacodynamic (PK/PD) characteristics for immunocompetent and immunocompromised patients; may depend on compound mechanism of action
• Assay standardization and provision of key assays as a service to enable comparison of compounds and distribution of resources
• Potential key assays:
○ In vitro phenotypic testing (e.g., IC50, rate-of-action and parasite elimination, stage-of-action)
○ Methods to determine the probability of resistance
○ Methods for target validation, to identify mechanism of action, and, if relevant, mechanism of resistance
○ Human challenge model, allowing early testing of candidates in adult volunteers

In addition to efficacy against a range of *C*. *parvum* and *C*. *hominis* isolates, an ideal candidate would also be curative at the same dose for other protozoan pathogens that cause diarrhea, since it might facilitate syndromic treatment in the absence of a cost-effective point-of-care diagnostic test. Based on several important caveats, however, we do not believe this should be the basis of go/no-go decision making for *Cryptosporidium* drug development. First, *Giardia intestinalis* infection, long accepted as a cause of diarrhea and a likely cause of malnutrition, appeared to protect against life-threatening childhood diarrhea in the GEMS study [1]. Thus, there is considerable uncertainty at present as to whether treatment of *Giardia* infection may sometimes actually be harmful. Low-level activity against other pathogens could also be detrimental by fostering resistance for those pathogens against other drugs working by the same mechanism. Nevertheless, accurate diagnosis is complicated by the frequent presence of multiple potential pathogens in the main target patient population (e.g., two or more agents were identified in 45% of cases in the GEMS study [1]), so a single medicine with activity against multiple pathogens and a high barrier to resistance for each would be more economically viable and have obvious clinical advantages.

A viable medicine must of course meet essential safety, dosing, cost, and stability standards. Despite the caveats noted above, syndromic therapy is typical for patients with diarrhea, so drug safety should ideally be adequate for treatment of all patient populations with diarrhea in the absence of a specific diagnosis of cryptosporidiosis. Drug interactions should be minimal. Given the desired use in HIV-positive patients who will hopefully be receiving highly active antiretroviral therapy (HAART), drugs should ideally not inhibit cytochrome P4503A4 (CYP3A4); however, the greatest need for treatment is in young, HIV-negative children, so lack of CYP3A4 inhibition should not be considered essential. The true global burden of cryptosporidiosis remains unknown, and the specific target countries for treatment availability will need to be adjusted as the epidemiology becomes clear. However, at a minimum, countries for which cryptosporidiosis is known to be endemic and other countries that contribute heavily to diarrhea-related mortality should be included. Given the supply chain in the developing world, which often passes through a centralized national medicines repository, new products must be stable for as long as possible under conditions of high temperature and humidity (Zone IVb [30°C and 75% relative humidity]) for a minimum of 2 years and ideally for 5 years. Dosing must be oral, minimally affected by food intake, and should be compatible with a hydrodispersible tablet or granules with appropriate taste masking for children. Ideally, a single dose would be curative, but given the absence of an effective therapy for chronic cryptosporidiosis, even dosing three times per day for 14 days could be acceptable for some patient populations. The cost should be comparable to or lower than that of nitazoxanide (in India currently ~$US0.50 in total for dosage of 100 mg/5 ml oral suspension for 3 days). For reference, the bulk price of nitazoxanide is currently US$50–US$100 per kilogram, or 0.5–1 US cent per gram, showing that most of the current costs are actually in the manufacturing of the tablets.

## Critical Unknowns and Closing Thoughts

There is an enormous opportunity to improve the lives of many of the world’s most vulnerable children by developing improved treatments and/or preventive measures for cryptosporidiosis. Accomplishing this goal will require both great science and a keen awareness of the end product desired. The target product characteristics and developmental cascade outlined above are based largely on common sense and on practical considerations that are typical for any drug development campaign and specific to the patient populations in need. There are many unknowns regarding how best to achieve these goals, which will likely require modification of this target product profile and compromises as progress is made. For example, the extent to which drug resistance may be the basis of treatment failures or develop de novo is not clear, and development of drug combinations in which each component has a different mechanism of action might be necessary to address this possibility. Based on the experience with malaria, methods to assess the likelihood of resistance may be important for logical compound prioritization and informative about the desirability of developing single agents versus combination therapies. Methods are also needed to validate potential drug targets and identify the mechanism of action for promising compounds (see Table 2). Finally, since *Cryptosporidium* parasites typically reside within intestinal epithelial cells in immunocompetent people, but can spread to involve the biliary tree epithelium and, rarely, even the respiratory epithelium in severely immunocompromised patients [49], it remains unclear if a single treatment can be developed to treat both immunocompetent children and immunocompromised patients.

The preclinical and clinical studies to identify a product with this TPP still need some refinement by the community (see Fig 1 and Table 2). The in vitro assays clearly require a range of parasites and a selectivity assay to show a therapeutic window compared with mammalian cells at an early stage. The minimum acceptable activity for an initial hit will be an important debate. For malaria, the threshold has been set at <1 μM. Increasing the threshold beyond this level is always tempting, since it increases the number of hits, but is often ill-advised because of the difficulty in advancing such series [50]. The animal models needed to validate such a series in vivo are more challenging. Initial data on pharmacokinetics are fundamentally important in order to determine if the molecule produces intestinal and plasma exposure after an oral dose that are consistent with having activity. However, at this time the optimal PK characteristics for treatment of cryptosporidiosis remain poorly defined; some studies have indicated that a high level of intestinal exposure is critical [33], while others have concluded differently [25]. The optimal PK characteristics may also depend on a compound’s mechanism of action, and due to spread of infection beyond the intestinal tract in immunocompromised individuals, may depend on the target patient population.

Malnourished mice provide a model to study weight loss and interactions of cryptosporidiosis with malnutrition [51,52]. Unfortunately, none of the murine *Cryptosporidium* infection systems provides an ideal drug screening system, as the mice do not develop diarrhea and the licensed product, nitazoxanide, is not active in most mouse–*Cryptosporidium* infection models, including malnourished mice [51–53]. The newborn calf–*Cryptosporidium parvum* challenge model is a clinical model with patent diarrhea, and the calf model does respond to nitazoxanide therapy, at least in the hands of some investigators [54]. However, a robust and inexpensive animal model to test compounds for activity against *C*. *hominis* is clearly needed.

Another uncertainty is the clinical development pathway, from adults with experimental *Cryptosporidium* challenge to children aged 1–23 months who appear to bear the brunt of *Cryptosporidium* infection. The key to facilitate progression to trials in young children is to have good safety data in otherwise healthy adults, since these represent the cleanest background against which adverse events can be assessed for anti-*Cryptosporidium* clinical trials when seeking regulatory approval for a pediatric indication. A clear focus on the first human clinical proof of concept is essential to any efficient drug discovery and development effort. The challenge is to achieve consensus for the minimal number of assays on the critical path to defining safe and active new clinical candidates. This TPP is based on our experience of *Cryptosporidium* infections on the one hand and malaria drug discovery and development on the other. The hope is that this template will be useful for streamlining the discovery and development of a new generation of products to reduce the morbidity and mortality caused by diarrhea.
